# Metabolic biomarkers of risperidone-induced weight gain in drug-naïve patients with schizophrenia

**DOI:** 10.3389/fpsyt.2023.1144873

**Published:** 2023-04-20

**Authors:** Yuying Qiu, Yeqing Dong, Wei Sun, Gang Li, Mei Juan Li, Yongping Zhao, Changyong Jiang, Jie Li

**Affiliations:** ^1^Laboratory of Biological Psychiatry, Institute of Mental Health, Tianjin Anding Hospital, Mental Health Center of Tianjin Medical University, Tianjin, China; ^2^Chifeng Anding Hospital, Inner Mongolia Autonomous Region, Chifeng, China

**Keywords:** schizophrenia, weight gain, risperidone, targeted metabolomics, biomarkers

## Abstract

**Background:**

Risperidone is a commonly prescribed antipsychotic drug with a potential side effect of weight gain. However, the pathophysiological mechanism is still poorly understood. Here, we sought to identify potential biomarkers of risperidone-induced weight gain by using a targeted metabolomics approach.

**Methods:**

We enrolled 30 subjects who received risperidone monotherapy for 8  weeks from a prospective longitudinal cohort study for drug-naïve schizophrenia patients. Plasma metabolites were measured by targeted metabolomics Biocrates MxP® Quant 500 Kit at baseline and 8-week follow-up.

**Results:**

After 8  weeks of risperidone treatment, the levels of 48 differential metabolites were upregulated, including lysophosphatidylcholines (2), phosphatidylcholines (PC) (8), cholesteryl esters (CE) (3), and triglycerides (35), while 6 differential metabolites namely PC aa C38:6, methionine (Met), α-aminobutyric acid (AABA), TrpBetaine, CE (22:6), and Taurocholic acid (TCA) were downregulated. Interestingly, the reduction of PC aa C38:6, AABA and CE (22:6) was linearly related with increased BMI. Further multiple regression analysis showed that the changes of PC aa C38:6 and AABA were independent contributors of increased BMI. In addition, baseline levels of PC aa C36:5, CE (20:5) and AABA had positive relationships with the change of BMI.

**Conclusion:**

Our findings indicate phosphatidylcholines and amino acids may serve as biomarkers for risperidone-induced weight gain.

## Introduction

Weight gain and obesity often occur in individuals treated with antipsychotics (1), which may heighten the likelihood of diabetes and cardiovascular disease and shorten life span (2). A meta-analysis reported a 3.22 kg mean weight gain and 1.4 kg/m^2^ BMI change within the first 12 weeks of antipsychotic treatment, and 5.30 kg and 1.86 kg/m^2^ in the long term in first-episode patients (3). Risperidone is the most commonly prescribed antipsychotic drug due to a broad efficacy for psychiatric disorders and lower cost for patients. Unfortunately, risperidone is also prone to cause significant weight gain in adolescents and adults (4–7).

Previous studies have focused on neurotransmitter pathways to reveal the mechanism of risperidone inducing weight gain, such as serotonin, histaminergic H1 receptors and neuropeptide Y in hypothalamic (8–10). However, the pathogenesis remains unclear. As a rapidly evolving field, metabolomics enables us to measure global changes in metabolic profiles of individuals in response to disease or drug treatment. It has been widely used for diagnosing diseases and assessing drug efficacy (11, 12). Current metabolomics studies in schizophrenia have focused on identifying biomarkers for diagnostic and therapeutic tools. For instance, an untargeted metabolomic profiling research based on gas chromatography–mass spectrometry suggested that tryptophan, uric acid, and myo-inositol might be potential serum biomarkers related to schizophrenia and risperidone treatment (13). Nevertheless, fewer studies have reported changes in metabolites associated with risperidone-induced weight gain.

In this study, we utilized targeted metabolomics Biocrates MxP® Quant 500 Kit to detect blood samples that collected from drug-naïve patients with schizophrenia at baseline and after 8 weeks of risperidone treatment. At last, 54 differential metabolites were preliminarily screened and further analyzed for pathway enrichment to search for potential weight gain relevant biomarkers.

## Methods

### Subjects

The samples were from a prospective longitudinal cohort study for drug-naïve patients with schizophrenia. Two hundred subjects were recruited from November 2017 to July 2020 in the inpatient departments of Tianjin Anding Hospital. Eligible patients met the following criteria: (1) Chinese Han; (2) aged 18–65 years; (3) diagnosis of schizophrenia or schizophreniform disorder according to Diagnostic and Statistical Manual of Mental Disorders, Fifth Edition (DSM-5); (4) never received antipsychotics treatment; and (5) total score of positive and negative symptom scale (PANSS) > 60. Exclusion criteria included: (1) alcoholism or other substance (other than tobacco) abuse or dependence; (2) severe physical diseases; and (3) pregnant or lactating women. As a natural observational study, patients received antipsychotic treatment at the discretion of their clinicians. In the present study, 30 of the 200 patients (15.0%) were treated for 8 weeks with risperidone monotherapy. During the 8-week follow-up, the daily dose of risperidone was based on the doctor’s judgment. The combinations of benzodiazepines and drugs for the extrapyramidal side effects were acceptable. But subjects treated with other antipsychotics, valproic acid, lithium carbonate, carbamazepine, or antidepressants were excluded. All the participants were hospitalized and on a uniform diet. The main exercises were doing gymnastics and walking organized by nurses for 30 min a day.

The study was approved by the Medical Ethics Committee of Tianjin Anding Hospital (Number 2017–03). All participants signed informed consent forms prior to participation.

### Clinical assessment and metabolic assessment

Clinical assessment and anthropometric data were obtained at baseline and 8 weeks. The severity of psychotic symptoms was assessed with the PANSS (14) by raters who were well-trained. We measured the height, body weight, waist circumference (WC), and hip circumference (HC) after an overnight fast of at least 8 h. Laboratory examinations including fasting plasma glucose (FPG), triglycerides (TG), high-density lipoprotein cholesterol (HDL-C), low-density lipoprotein cholesterol (LDL-C), and total cholesterol (TC) were also recorded.

### Sample collection

Subjects were fasted overnight before blood samples were obtained. The antecubital vein was collected as a baseline sample on the morning of day 0 of the study. The sample was also taken approximately 12 h after the last dose of risperidone at the end of 8 weeks. The plasma was frozen at-80°C until further analysis after centrifuging all blood samples at 1000 rpm for 15 min at 4°C.

### Sample preparation and FIA/LC–MS analysis

The targeted metabolomic analysis of plasma samples was carried out by tandem mass spectrometry with the Biocrates MxP® Quant 500 Kit (Biocrates, Innsbruck, Austria). Six hundred and thirty endogenous metabolites belonging to 26 biochemical classes, including amino acids, fatty acids, lysophosphatidylcholines, and phosphatidylcholines, have been quantified in the kit. A detailed description of experimental metabolomics measurement techniques can be found at https://patents.google.com/patent/ EP1875401B1 and https://patents.google.com/patent/ EP1897014B1.

In brief, sample preparation was conducted using a 96-well device to quantify metabolite profiles. In this device, inserts have been impregnated with internal standards. And, a predefined sample amount has been added. Next, some analytes were derivatized with a phenyl isothiocyanate (PITC) solution, and after derivatization, the analytes were extracted by organic solvents and then diluted. Finally, the extracts were examined by Liquid chromatography–mass spectrometry (LC–MS/MS) and Flow Injection Analysis Tandem Mass Spectrometry (FIA-MS/MS) methods *via* a 6,500 QTRAP® instrument (AB Sciex, Singapore).

### Statistical data analysis

Data were imported into Biocrates MetIDQ™ software for calculation of analyte concentrations, data evaluation, etc. All metabolite concentrations were calculated in ng/ml and normalized. Variables missed in 50% or greater of all samples were removed, and the rest missing measurements were substituted with the arithmetic mean of all valid measurements for further statistical analysis. SIMCA software (version 14.0, Umetrics, Umea, Sweden) was used for orthogonal partial least squares discriminant analysis (OPLS-DA) and variable importance in the projection (VIP) values were obtained. To evaluate the fitting of the OPLS-DA model, 100 permutation validations were carried out. According to the grouping of samples, T-tests were utilized to assess the significance of the variables. Differential metabolites were selected with VIP > 1 and *p* < 0.05. The heat map and pathway enrichment analysis of differential metabolites were performed based on MetaboAnalyst 5.0 platforms.^1^

SPSS 23.0 was utilized to analyze the clinical variables. Paired *T*-test was for the comparison of clinical variables before and after treatment. We used Pearson correlation to evaluate the relationship between the changes of anthropometric parameters (including BMI, HC and WC) and differential metabolites. A multiple linear regression analysis was used to assess the effects of several Independent variables, including age, sex, baseline BMI and the changes of differential metabolites on the change of BMI. Partial correlation analysis adjusting for age, sex, and baseline values to assess the effects of baseline levels of metabolites on the changes of anthropometric parameters (including BMI, HC and WC). A two-tailed *p* < 0.05 was considered statistically significant.

## Results

### Clinical characteristics of subjects

The present study was composed by 30 patients with schizophrenia (15 males and 15 females) who received risperidone monotherapy for 8 weeks. The average age was (40.47 ± 11.50) years and the average onset age was (36.40 ± 12.19) years. The shortest duration of illness was 1 month and the longest was 240 months, with an average of (51.77 ± 63.96) months. There were 7 patients who smoked and 7 patients with a family history of mental illness.

After 8-week treatment, 19 patients (63.33%) experienced weight gain, of them 4 patients (13.33%) with weight gain ≥7% and 11 patients (36.67%) with weight loss/unchanged. The mean change in body weight from baseline was 1.35 kg. The difference between baseline and 8 weeks follow-up was statistically significant (*p* < 0.05). Similar results were found in BMI, WC, and HC. But there were no significant differences in TG, TC, HDL-C, LDL-C, or FPG (*p* > 0.05). It was noteworthy that 3 of 30 patients (10.00%) developed metabolic syndrome (MetS) which diagnosed according to the National Cholesterol Education Program Adult Treatment Panel (NCEP-ATP) III guidelines (15).

After risperidone monotherapy for 8 weeks, the total PANSS score, positive symptoms, negative symptoms, and general psychopathology improved significantly (*p* < 0.001; Table 1).

**Table 1:** Anthropometric, metabolic and psychiatric outcomes in drug-naive patients with schizophrenia at baseline and after 8 weeks of treatment with risperidone.

	Baseline (*n* = 30)	8 weeks (*n* = 30)	*t*	*p*
Body weight (kg)	63.55 ± 12.24	64.90 ± 11.80	−2.833	0.008
BMI(kg/m^2^)	22.85 ± 3.22	23.34 ± 3.09	−2.917	0.007
WC (cm)	80.57 ± 8.11	81.60 ± 8.11	−2.868	0.008
HC (cm)	91.83 ± 8.10	93.13 ± 8.19	−3.556	0.001
TC (mmol/L)	4.60 ± 1.02	4.46 ± 1.01	1.049	0.303
TG (mmol/L)	1.27 ± 0.70	1.67 ± 1.09	−1.827	0.078
LDL-C (mmol/L)	2.74 ± 0.73	2.58 ± 0.81	1.559	0.130
HDL-C (mmol/L)	1.41 ± 0.42	1.33 ± 0.36	1.304	0.203
FPG (mmol/L)	4.84 ± 0.74	4.59 ± 0.56	1.739	0.093
PANSS Total score	87.53 ± 12.63	58.53 ± 12.82	12.488	<0.001
Positive symptoms score	25.47 ± 3.48	13.97 ± 4.83	15.523	<0.001
Negative symptoms score	19.87 ± 6.40	16.00 ± 4.62	4.312	<0.001
General psychopathology score	42.17 ± 7.43	28.40 ± 6.48	9.462	<0.001

BMI, Body mass index; WC, Waist circumference; HC, Hip circumference; FPG, fasting plasma glucose; TG, Triglyceride; TC, Total Cholesterol; HDL-C, high-density lipoprotein cholesterol; LDL-C, low-density lipoprotein cholesterol.

### Quality control

QC samples with known concentrations were inserted into the cohort of samples, and underwent the same pre-treatment and mass spectrometry detection process as the samples to be tested. The precision of QC samples demonstrated the quality and stability of the data throughout the analytical run (Supplementary Figure S1).

### Metabonomics analysis at baseline and after 8-week treatment with risperidone

**Figure 1:**
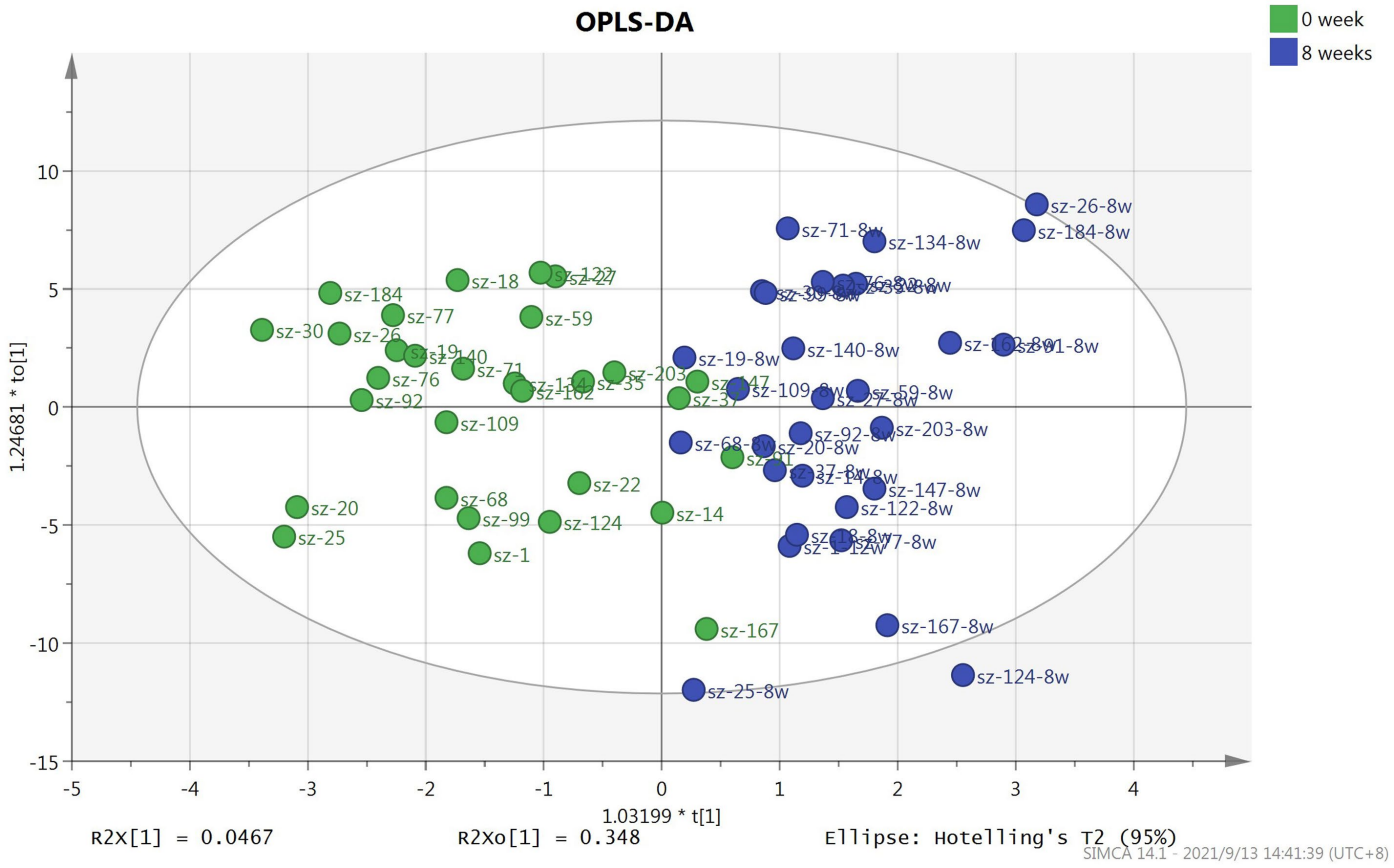
Score plot of OPLS-DA model based on plasma samples for patients with schizophrenia at baseline and after 8-week treatment with risperidone.

In this study, a total of 291 metabolites were quantified by FIA/LC–MS. By utilizing SIMCA software, the OPLS-DA model was developed to evaluate metabolic differences between plasma at baseline and after 8 weeks of risperidone therapy. The results of the score plot showed that the OPLS-DA model achieved a clear discrimination between the baseline and 8-week follow-up (Figure 1). Permutation tests of 100 times were conducted on the supervised models (Supplementary Figure S2). Among these compounds, we found 132 differential compounds with VIP > 1 and 89 compounds with *p* < 0.05. According to the differential variables screening criteria of VIP > 1 and *p* < 0.05, 64 metabolites were identified to distinguish between before and after risperidone treatment. Further combined with fold change >1.2, 54 differential metabolites were obtained, mainly including amino acids, phosphatidylcholines, triglycerides, etc. Among them, the concentration levels of 48 differential metabolites were upregulated, while 6 were downregulated after 8 weeks of risperidone treatment (Table 2).

**Table 2:** Differential metabolites in drug-naive patients with schizophrenia at baseline and after 8 weeks of treatment with risperidone.

Metabolites	VIP^a^	*p* ^b^	FC^c^	Related pathway (KEGG)
TG(16:0_32:1)	2.1738	0.0267	0.4649	Fat digestion and absorption, lipid and atherosclerosis and others
TG(16:0_32:0)	1.8741	0.0351	0.4803	Fat digestion and absorption, lipid and atherosclerosis, and others
TG(18:0_32:1)	1.4750	0.0265	0.4834	Fat digestion and absorption, lipid and atherosclerosis, and others
TG(16:0_32:2)	1.9219	0.0232	0.5518	Fat digestion and absorption, lipid and atherosclerosis, and others
TG(14:0_34:1)	1.9168	0.0308	0.5632	Fat digestion and absorption, lipid and atherosclerosis, and others
TG(20:4_32:0)	1.6907	0.0206	0.5815	Fat digestion and absorption, lipid and atherosclerosis, and others
TG(18:2_30:0)	1.9477	0.0210	0.5848	Fat digestion and absorption, lipid and atherosclerosis, and others
TG(16:0_32:3)	1.6722	0.0149	0.5876	Fat digestion and absorption, lipid and atherosclerosis, and others
TG(18:1_30:1)	1.6407	0.0440	0.5920	Fat digestion and absorption, lipid and atherosclerosis and others
TG(18:1_30:0)	1.8248	0.0473	0.5932	Fat digestion and absorption, lipid and atherosclerosis, and others
TG(18:0_34:2)	1.6624	0.0221	0.5965	Fat digestion and absorption, lipid and atherosclerosis and others
TG(20:5_36:2)	1.0821	0.0251	0.6037	Fat digestion and absorption, lipid and atherosclerosis, and others
TG(14:0_34:2)	1.7946	0.0377	0.6046	Fat digestion and absorption, lipid and atherosclerosis and others
TG(18:3_36:1)	1.0460	0.0416	0.8015	Fat digestion and absorption, lipid and atherosclerosis and others
TG(18:0_30:0)	1.7872	0.0138	0.6151	Fat digestion and absorption, lipid and atherosclerosis, and others
TG(18:3_32:0)	1.7450	0.0233	0.6161	Fat digestion and absorption, lipid and atherosclerosis and others
TG(16:0_34:0)	1.4839	0.0313	0.6182	Fat digestion and absorption, lipid and atherosclerosis, and others
TG(18:1_30:2)	1.8941	0.0174	0.6311	Fat digestion and absorption, lipid and atherosclerosis, and others
TG(16:0_35:3)	2.0246	0.0177	0.6520	Fat digestion and absorption, lipid and atherosclerosis, and others
TG(18:3_30:0)	1.5491	0.0295	0.6532	Fat digestion and absorption, lipid and atherosclerosis, and others
TG(16:0_35:2)	1.4426	0.0458	0.6643	Fat digestion and absorption, lipid and atherosclerosis, and others
TG(20:4_32:1)	1.2046	0.0476	0.6719	Fat digestion and absorption, lipid and atherosclerosis, and others
TG(16:0_34:4)	1.7228	0.0190	0.6744	Fat digestion and absorption, lipid and atherosclerosis, and others
TG(18:2_30:1)	1.2550	0.0299	0.6833	Fat digestion and absorption, lipid and atherosclerosis, and others
TG(18:0_36:1)	1.2892	0.0186	0.6888	Fat digestion and absorption, lipid and atherosclerosis, and others
TG(18:3_32:1)	1.4775	0.0285	0.7088	Fat digestion and absorption, lipid and atherosclerosis, and others
TG(18:0_34:3)	1.1835	0.0498	0.7099	Fat digestion and absorption, lipid and atherosclerosis, and others
TG(16:0_38:2)	1.6928	0.0391	0.7105	Fat digestion and absorption, lipid and atherosclerosis, and others
TG(16:0_36:5)	1.4209	0.0154	0.7124	Fat digestion and absorption, lipid and atherosclerosis, and others
TG(20:5_34:1)	1.1804	0.0066	0.7241	Fat digestion and absorption, lipid and atherosclerosis, and others
TG(16:0_38:4)	1.3349	0.0262	0.7377	Fat digestion and absorption, lipid and atherosclerosis, and others
TG(18:2_36:0)	1.1839	0.0026	0.7452	Fat digestion and absorption, lipid and atherosclerosis, and others
TG(18:2_35:1)	1.1741	0.0408	0.7673	Fat digestion and absorption, lipid and atherosclerosis, and others
TG(18:3_34:0)	1.0754	0.0269	0.7685	Fat digestion and absorption, lipid and atherosclerosis, and others
TG(18:3_34:1)	1.3274	0.0443	0.7769	Fat digestion and absorption, lipid and atherosclerosis, and others
CE(22:6)	1.1891	0.0241	1.2042	Lipid and atherosclerosis, cholesterol metabolism, and others
CE(20:5)	1.1395	0.0221	0.7851	Lipid and atherosclerosis, cholesterol metabolism, and others
CE(14:0)	1.8279	0.0175	0.6105	Lipid and atherosclerosis, cholesterol metabolism and others
CE(18:3)	1.9327	0.0030	0.6891	Lipid and atherosclerosis, cholesterol metabolism, and others
PC aa C40:5	1.1233	0.0045	0.7778	Glycerophospholipid metabolism, arachidonic acid metabolism, and others
PC aa C38:6	1.2544	0.0002	1.2200	Glycerophospholipid metabolism, arachidonic acid metabolism, and others
PC aa C34:4	1.5263	0.0028	0.7901	Glycerophospholipid metabolism, arachidonic acid metabolism, and others
PC aa C36:5	1.2118	0.0015	0.7915	Glycerophospholipid metabolism, arachidonic acid metabolism, and others
PC aa C32:2	1.5243	0.0157	0.7976	Glycerophospholipid metabolism, arachidonic acid metabolism, and others
PC aa C38:3	1.2017	0.0078	0.8014	Glycerophospholipid metabolism, arachidonic acid metabolism, and others
PC aa C32:1	1.4076	0.0173	0.7231	Glycerophospholipid metabolism, arachidonic acid metabolism, and others
PC aa C40:4	1.2687	0.0016	0.7460	Glycerophospholipid metabolism, arachidonic acid metabolism, and others
PC aa C34:3	1.3488	0.0087	0.8277	Glycerophospholipid metabolism, arachidonic acid metabolism, and others
lysoPC a C20:3	1.1671	0.0060	0.8192	Glycerophospholipid metabolism
lysoPC a C14:0	1.9853	0.0003	0.6825	Glycerophospholipid metabolism
Met	1.0990	0.0001	1.2011	Cysteine and methionine metabolism, metabolic pathways, and others
AABA	1.9201	0.0008	1.5340	Cysteine and methionine metabolism and metabolic pathways
TCA	1.0035	0.0400	1.4044	Primary bile acid biosynthesis, Taurine and hypotaurine metabolism, and others
TrpBetaine	1.1658	0.0000	1.5488	-

Variable importance in the projection (VIP) values were obtained from OPLS-DA models with a threshold of 1; p value was calculated from T-test with a threshold of 0.05; Fold change (FC) was calculated as the ratio of the mean metabolite levels between two groups.

### Clustering and pathway enrichment analysis

**Figure 2:**
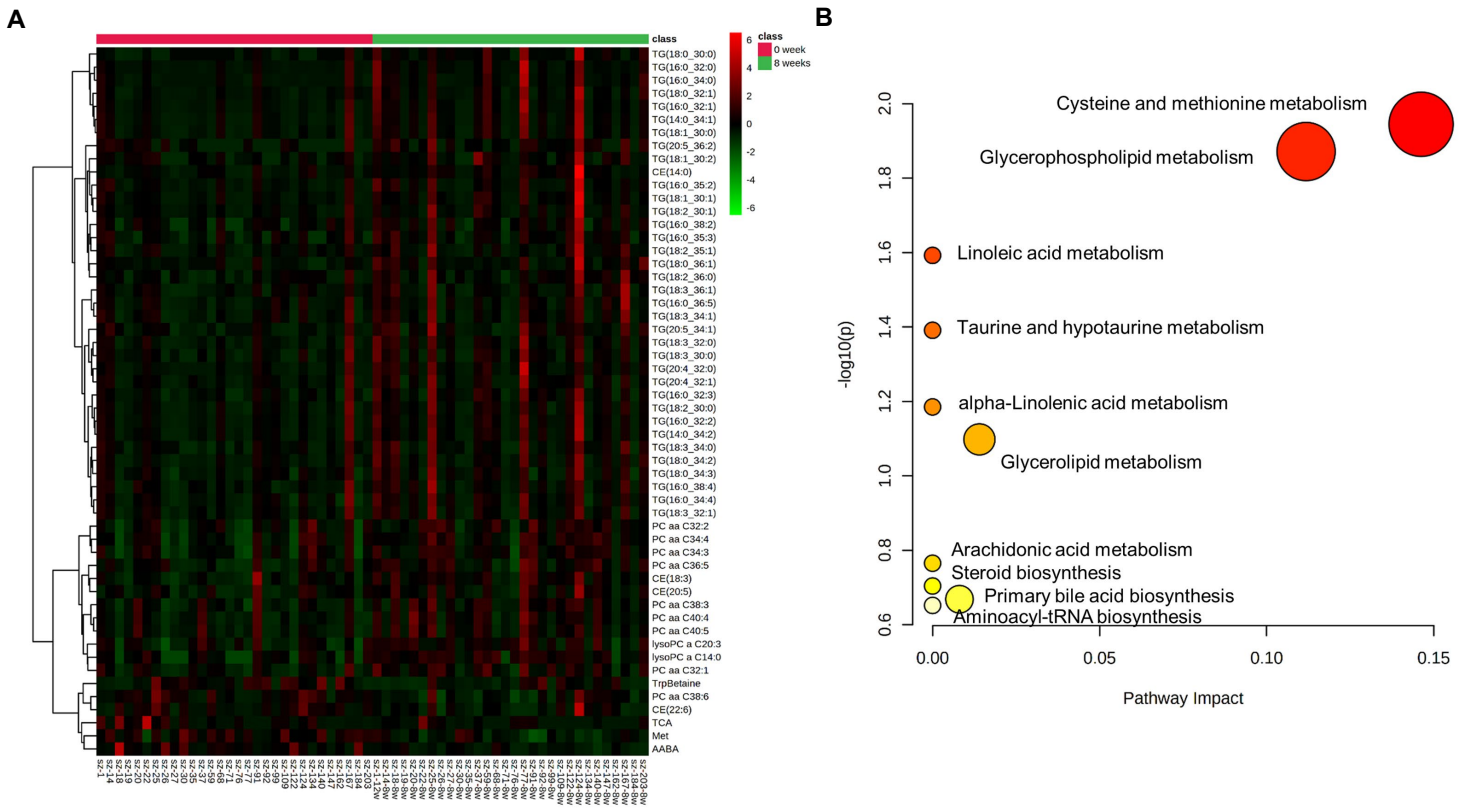
Clustering and pathway enrichment analysis of differential metabolites. **(A)** Heat map of differential compounds by clustering analysis according to their variation trends. **(B)** Pathway enrichment analysis of differential metabolites. The *p* value and pathway impact values were calculated from the enrichment analysis and pathway topology analysis, respectively.

Based on MetaboAnalyst platform, the 54 differential metabolites were further analyzed for clustering and pathway enrichment analysis. The results suggested that these compounds were mainly divided into 3 clusters (Figure 2A). The differential metabolites in Cluster I was composed of TG, such as TG (18:0_30:0), TG (16:0_32:1) and others, whose concentration increased after 8 weeks of risperidone treatment. The metabolites of Cluster II mainly include phosphatidylcholines, lysophosphatidylcholines, and cholesteryl esters, the concentration of which was also significantly increased. Moreover, Cluster III consisted of TrpBetaine, PC aa C38:6, CE (22:6), TCA, Met and AABA and its concentration was significantly decreased after risperidone treatment. As shown in Figure 2B, there were three crucial metabolic pathways, cysteine and methionine metabolism, glycerophospholipid metabolism, and linoleic acid metabolism, were significantly enriched in differential metabolites of schizophrenic patients after 8-week risperidone treatment compared to the baseline. Of these, cysteine and methionine metabolism pathway was most affected.

### Correlation analysis between differential metabolites and weight gain

Pearson correlation analysis indicated that the change of BMI was negatively correlated with the changes of CE (22:6) (*r* = −0.490, *p* = 0.006), AABA (*r* = −0.523, *p* = 0.003) and PC aa C38:6 (*r* = −0.517, *p* = 0.003). Only the change of PC aa C38:6 had a negative relationship with the change of HC (*r* = −0.528, *p* = 0.003). However, no correlation was found between the changes of WC and metabolites. Based on multiple linear regression analysis, the changes of PC aa C38:6 (*β* = −0.367, *t* = −2.241, *p* = 0.033) and AABA (*β* = −0.378, *t* = −2.305, *p* = 0.029) had significant effects on BMI changes.

Specifically, partial correlation analysis adjusting for age, sex, and baseline values showed that baseline levels of PC aa C36:5, CE(20:5), and AABA were positively correlated with the change of BMI (*r_1_* = 0.392, *p_1_* = 0.043; *r_2_* = 0.417, *p_2_* = 0.031; *r_3_* = 0.416, *p_3_* = 0.031; Table 3). We also found that baseline levels of PC aa C36:5 were positively correlated with the change of HC (*r* = 0.383, *p* = 0.048), but no correlation was found in WC and metabolites.

**Table 3:** The correlation between the changes of BMI, HC, and differential metabolites.

Metabolites	The changes of BMI		The changes of HC	
	*r*	*p*	*r*	*p*
The changes of PC aa C38:6	−0.517	0.003	−0.528	−0.528
The changes of CE (22:6)	−0.490	0.006	−0.243	0.197
The changes of AABA	−0.523	0.003	−0.350	0.058
The baseline levels of PC aa C36:5	0.392	0.043^a^	0.383	0.048^a^
The baseline levels of CE (20:5)	0.417	0.031^a^	0.301	0.127^a^
The baseline levels of AABA	0.416	0.031^a^	0.326	0.097^a^

BMI, Body mass index; HC, Hip circumference; PC, Phosphatidylcholines; CE, Cholesteryl esters; AABA, α-aminobutyric acid.^a^Partial correlation analysis adjusted for age, sex, and baseline values.

## Discussion

Individuals treated with risperidone have a marked propensity to appear metabolic adverse, especially weight gain and obesity (16, 17). It may be a promising drug to elucidate the mechanism underlying weight gain induced by antipsychotics. Based on these considerations, we assessed the metabolite changes after the 8-week treatment of risperidone monotherapy in antipsychotic-naïve patients with schizophrenia by the Biocrates MxP® Quant 500 Kit following a targeted metabolomics approach.

In the longitudinal study, all patients were hospitalized at the 8-week follow-up which minimized confounding factors. We found that risperidone treatment significantly increased the weight of the patients. The mean weight gain was 1.35 kg, but these patients showed great variability, and the maximum gain 9 kg and maximum loss 4 kg. Our results were consistent with previous studies (18, 19), showing that risperidone-induced weight gain occurred in the early stage of treatment. Consistent with previous studies, we have found no significant changes in glucolipid metabolism parameters (20). Of note, the average value of TG, from 1.27 mmol/l (0.70 mmol/l) to 1.67 mmol/l (1.09 mmol/l), showed an upward trend, which suggested that abnormal glucose and lipid metabolism may occurred later than the side effects of weight gain during risperidone treatment.

The present study revealed that 54 differential metabolites were significantly altered in schizophrenia patients treated with risperidone for 8 weeks compared to baseline. Of them, 48 differential metabolites levels were upregulated, including lysophosphatidylcholines (2), phosphatidylcholines (8), cholesteryl esters (3), and triglycerides (35), while 6 differential metabolites, namely PC aa C38:6, Met, AABA, TrpBetaine, CE (22:6), and TCA, were downregulated. Further analysis showed that these compounds were mainly divided into 3 clusters, which were TG and amino acid-related and phosphatidylcholines-related metabolites. Numerous studies have shown that phospholipids played a key role in the function and structure of membranes and phospholipids metabolism abnormalities occurred in patients with schizophrenia treated with antipsychotics, including the upregulated phosphatidylcholines classes (21–23). A metabolomic mapping of atypical antipsychotic effects showed that risperidone treatment increased phosphatidylcholines concentrations (24). Lysophosphatidylcholines is the degraded product from phosphatidylcholines. Subjects being treated with the atypical drug, including risperidone, showed an increase in lysophosphatidylcholines compared to their baseline levels (24, 25), which support our results. However, it is worth mentioning that PC aa C38:6, which belongs to phosphatidylcholines, was significantly decreased after risperidone treatment in our study. This may be due to the different distribution of C-1 and C-2 positions of fatty acids with different lengths and saturation in glycerophosphate, leading to the different performance of the metabolite. Moreover, elevated TG levels may be the earliest and most sensitive indicator of the metabolic abnormalities associated with antipsychotics (26). Studies demonstrated the treatment with risperidone also elevated concentrations of TG (24, 27), which is consistent with our results. Previous studies have reported the roles of amino acid metabolism perturbation (28), phospholipid abnormalities (29), fatty acids alterations (30), and neurosteroid biosynthesis dysregulation (31) in the pathophysiology of schizophrenia. Our findings indicated that metabolic pathways, including cysteine and methionine metabolism, glycerophospholipid metabolism, and linoleic acid metabolism, were disturbed after 8-week risperidone treatment. Among them, especially cysteine and methionine metabolism changed the most. It suggests that risperidone treatment in patients with schizophrenia may exert regulatory effects on disorders caused by metabolic abnormalities, such as certain amino acids or phospholipids.

Previous metabonomics studies imply that phosphatidylcholines and lysophosphatidylcholines were closely related to overweight/obesity (32, 33). Phosphatidylcholines, as a major structural component of biological membranes, involved in some of the most fundamental metabolic activity of the organism (34, 35), such as promoting fatty acid oxidation, and reducing cholesterol or fatty acid absorption from the gastrointestinal tract (36). Administrated phosphatidylcholines has been shown to prevent lipid accumulation and body weight gain (37). Consisted with previous studies, we found a significant correlation between weight gain and phosphatidylcholines. In particular, the change of BMI was significantly negatively correlated with the change of PC aa C38:6, which indicated that phosphatidylcholines has a crucial role in weight gain induced by risperidone. Further regression analysis suggested that PC aa C38:6 might be used as a potential biomarker in weight gain after risperidone treatment. In terms of lysophosphatidylcholines, a metabolic profiling research suggested that higher levels of lysoPC C14:0 were in overweight/ obese subjects (38). Moreover, Liu et al. reported that the increased levels of LysoPC (14:0) was related to olanzapine-induced weight gain in female schizophrenia patients (39). Our study showed that LysoPC (14:0) and lysoPC a C20:3 were upregulated after 8 week treatment, but there was no association between weight gain and lysophosphatidylcholines, possibly because we also included male patients and there may be sex differences in metabolites. Olanzapine and risperidone may have different effects on lysophosphatidylcholines, which may be another reason for the discrepancy.

Studies have reported that plasma amino acids were closely correlated with obesity and metabolic dysfunction (40, 41). Determination of peripheral amino acid profile has been thought to identify visceral obesity (42). The amino acids metabolites such as serine, glycine, alanine and glutamate were decreased in patients with weight regain after Roux-en-Y gastric bypass which is one of the treatment for obesity (43). Plasma valine, leucine, isoleucine and AABA showed an early increase followed by a constant fall in obese children during hypocaloric dieting (44). We found that the concentrations of AABA reduced in the early stage of risperidone treatment. Pearson correlation analysis showed the change of AABA had a negative correlation with the change of BMI. These findings suggested that AABA might be a potential involvement in weight gain induced by risperidone.

In addition, we also examined the correlations between the baseline metabolite levels and the change of BMI, the results indicated that the higher baseline levels of PC aa C36:5, CE (20:5) and AABA in drug-naive patients with schizophrenia, the higher degree of weight gain. This finding suggests that these metabolites can be used as biomarkers to predict weight gain after risperidone treatment.

The present study has some limitations. First, although the patient’s diet was served by the nutrition canteen of our hospital, the amount of food intake and daily activity was not recorded. This might affect body weight and metabolites. Second, the relatively small sample size which reduced the statistical power. Third, as previous reported, clinically significant weight gain is defined as an increasing ≥7% of their baseline body weight (45). In our study, only 4 patients experienced weight gain ≥7% after 8 weeks of risperidone treatment, more significant weight gain will be better to clarify the findings. In addition, we did not consider the sex differences in hormones which more likely to influence the change of metabolites.

## Conclusion

In summary, our study identifies that PC aa C38:6 and AABA might be particularly involved in the biological mechanism of weight gain after risperidone treatment. In addition, baseline levels of PC aa C36:5, CE (20:5), and AABA may be predictors of weight gain caused by risperidone. Our findings might provide preliminary insight into the underlying mechanism of weight gain after taking risperidone in drug-naïve patients with schizophrenia. Further exploration and verification of these biomarkers in a larger cohort would be of great significance.

## Data availability statement

The datasets presented in this article are not readily available because it involves the personal privacy of the subjects and copyright for further use of data. Requests to access the datasets should be directed to corresponding author.

## Ethics statement

The studies involving human participants were reviewed and approved by the Ethical Committee of Tianjin Anding Hospital. The patients/participants provided their written informed consent to participate in this study.

## Author contributions

JL designed the study and wrote the study protocol, and manuscript preparation. YQ, YD, and WS recruited the patients, performed the statistical analyzes, and drafted the initial manuscript. GL, ML,YZ, and CJ performed the clinical rating and collecting the clinical data. All authors contributed to the final drafting and critically revised the manuscript.

## Funding

This work was reported by Tianjin Key Medical Discipline (Specialty) Construction Project, Award Number:TJYXZDXK-033A; Tianjin Key Medical Discipline (Specialty) Construction Project of Tianjin Health Commission, Award Number:TJWJ2022XK039; and Tianjin Health Commission Project, Award Number: KJ20067.

## Conflict of interest

The authors declare that the research was conducted in the absence of any commercial or financial relationships that could be construed as a potential conflict of interest.

## Publisher’s note

All claims expressed in this article are solely those of the authors and do not necessarily represent those of their affiliated organizations, or those of the publisher, the editors and the reviewers. Any product that may be evaluated in this article, or claim that may be made by its manufacturer, is not guaranteed or endorsed by the publisher.

## Footnotes

^1^ http://www.metaboanalyst.ca
