# Supplementary material for: Metabolic biomarkers of risperidone-induced weight gain in drug-naïve patients with schizophrenia
Source: Front Psychiatry. 2023 Apr 20;14:1144873. doi: 10.3389/fpsyt.2023.1144873 (PMC10171109; doi:10.3389/fpsyt.2023.1144873)
Supplement: Supplementary file 1 [file Data_Sheet_1.docx]

Supplementary Material

Metabolic biomarkers of risperidone-induced weight gain in drug-naïve patients with schizophreniae

Yuying Qiu^1 †^, Yeqing Dong^1 †^, Wei Sun^1 †^, Gang Li^1, 2^, Mei juan Li^1^, Yongping Zhao^1^, Changyong Jiang^1^ and Jie Li^1*^

*** Correspondence:** Jie Li: [jieli@tjmhc.com](mailto:jieli@tjmhc.com)

## Supplementary Figures

**
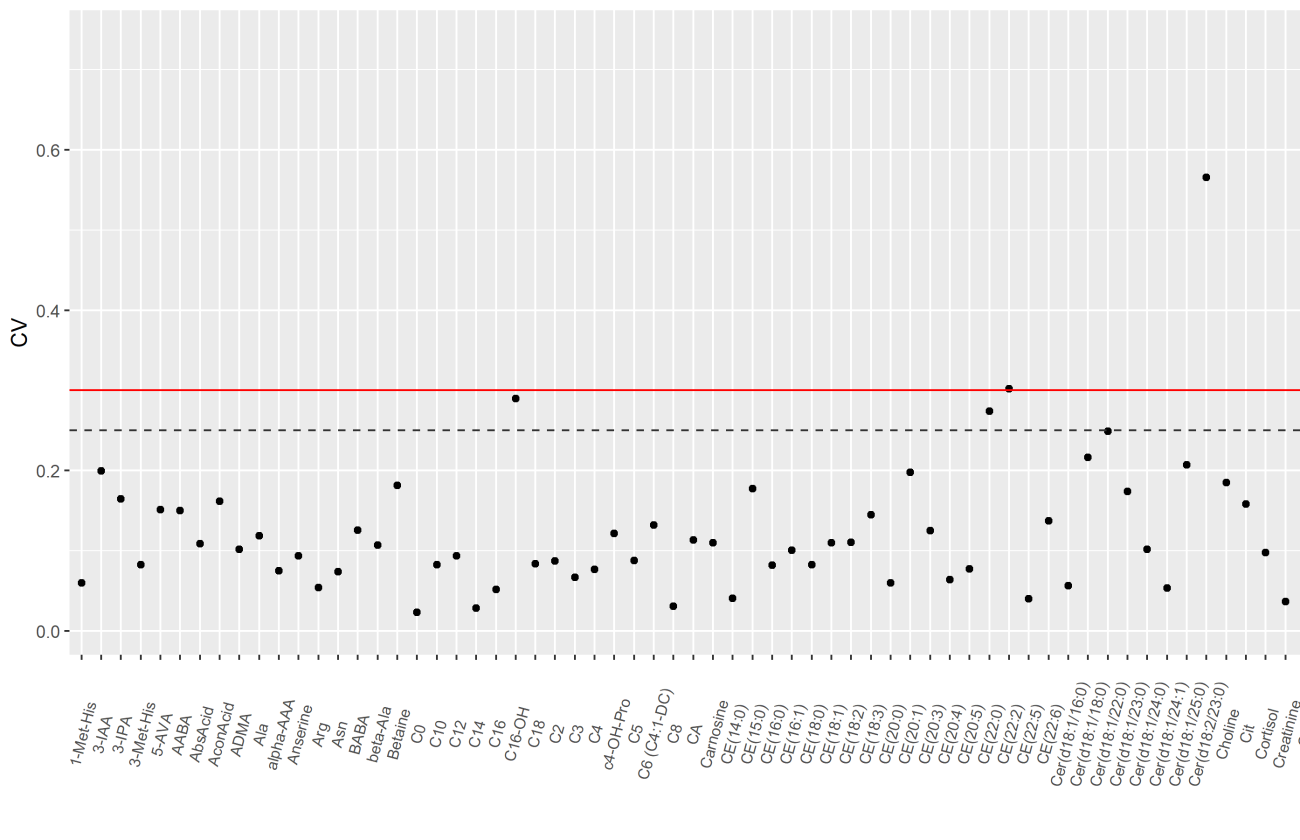
**

**Supplementary Fig.1** Stability assessment of analysis platform. The precision of some targets in QC samples showed that the precision of most metabolites was < 25%, indicating good stability of the analysis process.


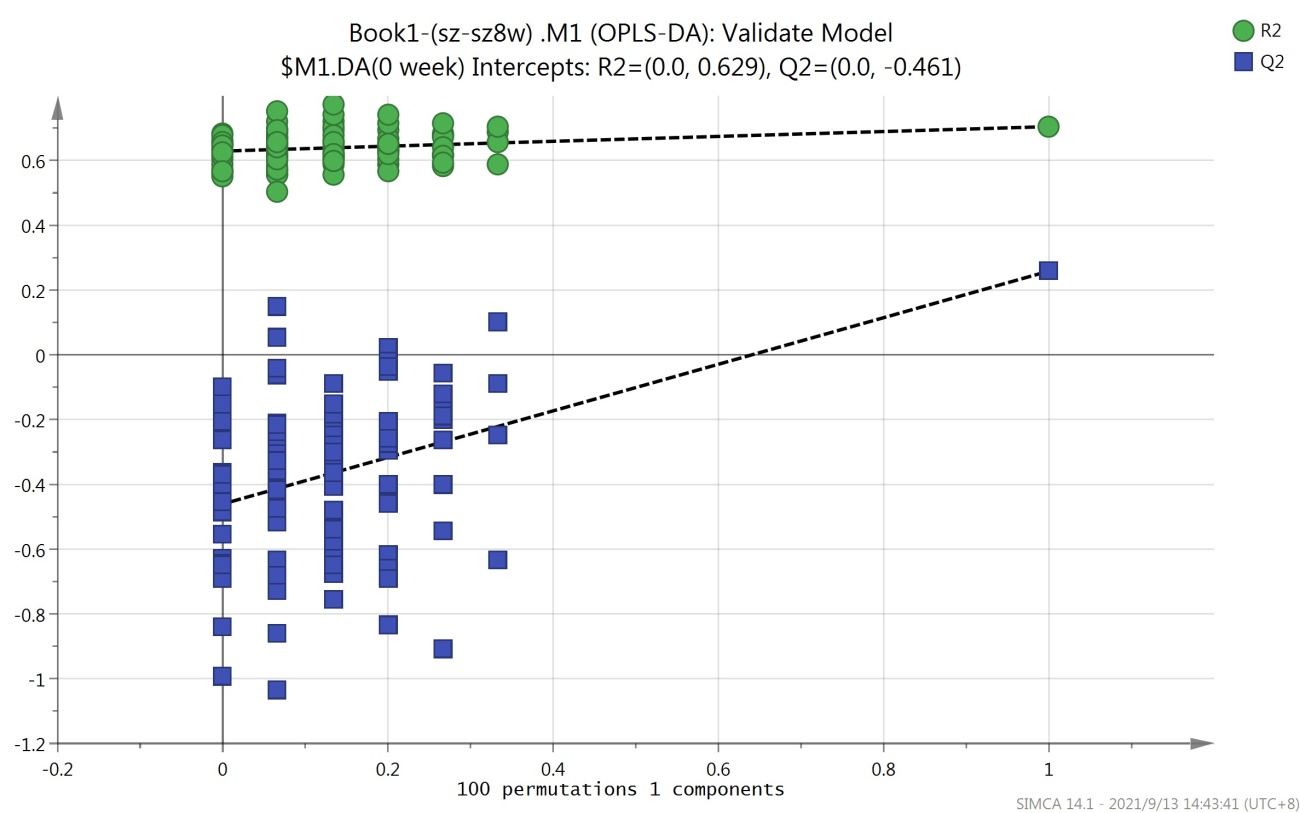


**Supplementary Fig.2** 100 permutation tests of the OPLS-DA model based on plasma samples for SZ patients at baseline and after 8-week treatment with risperidone groups. All blue Q2-values to the left are lower than the original points to the right. And the blue regression line of the Q2-points intersects the vertical axis (on the left), indicated no over fitting of OPLS-DA model.
